# Maternal–Fetal Implications of Mpox Infection: Current Evidence

**DOI:** 10.3390/jcm15010399

**Published:** 2026-01-05

**Authors:** Stefany Silva Pereira, Antonio Braga, Beatriz Bussi Rosolen, Talita Almeida Durães, Marcela Fermoselle de Vita Silva, Giovanna Alves de Britto, Giuliana Augustinelli Sales, Gustavo Yano Callado, Camilla Martins dos Santos Maia, Evelyn Traina, Edward Araujo Júnior, Gabriele Tonni, Roberta Granese

**Affiliations:** 1 Discipline of Woman Health, Municipal University of São Caetano do Sul (USCS), São Caetano do Sul 09521-160, SP, Brazil; stefany.pereira@uscsonline.com.br (S.S.P.); beatriz.rosolen@uscsonline.com.br (B.B.R.); talita.duraes@uscsonline.com.br (T.A.D.); marcela.silva1@uscsonline.com.br (M.F.d.V.S.); giovanna.britto@uscsonline.com.br (G.A.d.B.); giuliana.sales@uscsonline.com.br (G.A.S.); araujojred@terra.com.br (E.A.J.); 2 Department of Gynecology and Obstetrics, School of Medicine, Federal University of Rio de Janeiro (UFRJ), Rio de Janeiro 22240-003, RJ, Brazil; bragamed@yahoo.com.br; 3 Department of Maternal and Child Health, School of Medicine, Fluminense Federal University (UFF), Niterói 24070-090, RJ, Brazil; 4 Postgraduate Program in Applied Health Sciences, University of Vassouras, Vassouras 27700-000, RJ, Brazil; 5 Albert Einstein Israelite College of Health Sciences (FICSAE), Albert Einstein Israelite Hospital, São Paulo 05652-900, SP, Brazil; gycallado@gmail.com; 6 Department of Obstetrics, Paulista School of Medicine, Federal University of São Paulo (EPM-UNIFESP), São Paulo 04023-062, SP, Brazil; camilla.maia@unifesp.br (C.M.d.S.M.); etraina@unifesp.br (E.T.); 7 Department of Obstetrics and Neonatology, Istituto di Ricovero e Cura a Carattere Scientifico (IRCCS), AUSL Reggio Emilia, 42123 Reggio Emilia, Italy; gabriele.tonni@ausl.re.it; 8 Department of Biomedical and Dental Sciences and Morphofunctional Imaging, “G. Martino” University Hospital, 98100 Messina, Italy

**Keywords:** mpox, monkeypox virus, transmission, diagnosis, maternal–fetal outcomes, vaccine

## Abstract

Mpox is an emerging zoonotic infection caused by the Monkeypox virus, an Orthopoxvirus with increasing global relevance following the 2022 multinational outbreak. Historically endemic to Central and West Africa, the disease has evolved from sporadic zoonotic transmission to sustained human-to-human spread, particularly through close physical and intimate contact. Clinical manifestations typically include fever, lymphadenopathy, and progressive mucocutaneous lesions, although severity varies according to viral clade, immune status, and comorbidities. The 2022 outbreak, predominantly associated with the Clade IIb variant, was characterized by milder disease, localized lesions, and reduced mortality compared with the more virulent Clade I variant. Despite this, severe outcomes remain possible, particularly in vulnerable groups such as children, pregnant individuals, immunocompromised patients, and persons with extensive dermatological disorders. Diagnosis relies primarily on polymerase chain reaction testing from lesion-derived samples, with genomic sequencing serving as a complementary tool for epidemiological surveillance. Management is largely supportive, though antivirals such as tecovirimat may be considered in severe cases or in high-risk populations. Data regarding therapeutic safety in pregnancy are limited; however, tecovirimat appears to have the most favorable profile, whereas cidofovir and brincidofovir remain contraindicated. Prevention strategies include targeted vaccination with the non-replicating Modified Vaccinia Ankara–Bavarian Nordic vaccine, used for both pre- and post-exposure prophylaxis, particularly in individuals at elevated risk. Given the evolving epidemiological profile, the potential for vertical transmission, and the risk of adverse perinatal outcomes, Mpox infection during pregnancy poses unique clinical challenges. This review synthesizes current evidence on virology, clinical presentation, diagnosis, prevention, and management, with an emphasis on obstetric considerations and public health implications.

## 1. Introduction

**Figure 1:**
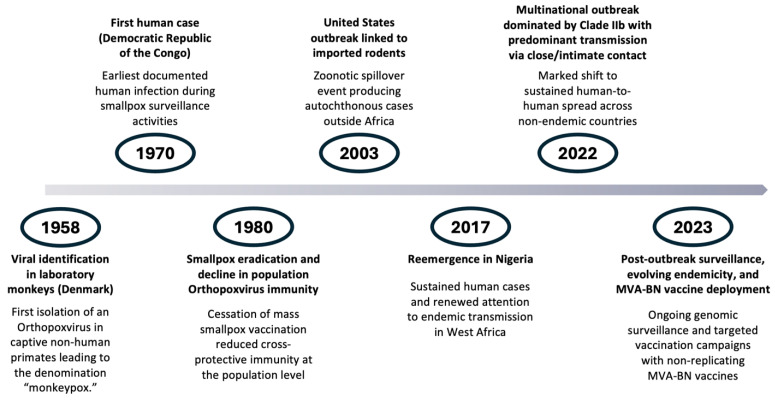
Epidemiological timeline of Mpox.

**Figure 2:**
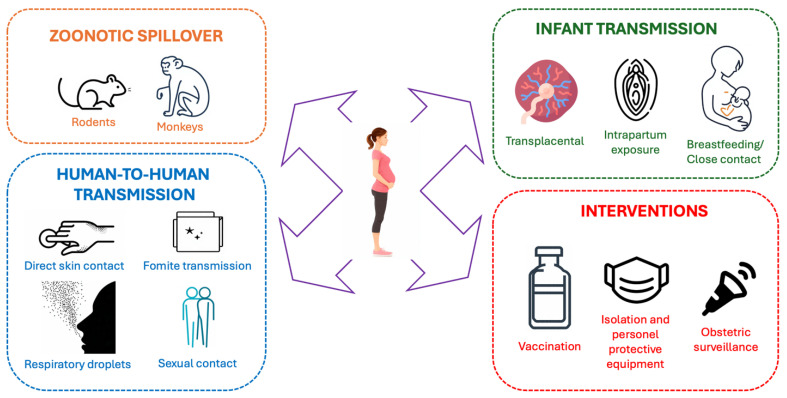
Transmission cycle of Mpox with obstetric routes.

Mpox is an infection caused by the Monkeypox virus, which belongs to the genus Orthopoxvirus and the family Poxviridae. It is a viral zoonosis first identified in laboratory monkeys in Denmark in 1958; however, the first human case was reported in 1970 in the Democratic Republic of the Congo, in Africa (Figure 1). Contrary to what its etymology might suggest, non-human primates are not the natural reservoirs of the disease, and transmission does not occur through direct contact with these animals. Instead, infection results from contact with rodents, infected individuals, and contaminated materials through lesions, body fluids, and respiratory droplets [1,2,3] (Figure 2).

Initially, Mpox was restricted to Central and West Africa, where it was endemic and associated with contact with wild animals carrying the virus. However, in 2022, a globally disseminated outbreak emerged, marking a shift in the disease’s pattern, as it began to affect countries such as the United States, Brazil, and European nations and became primarily transmitted through human-to-human contact, particularly among young adults aged 25 to 45 with active sexual lives in urban centers [4,5,6,7].

Because these viruses belong to the same family and share similar deoxyribonucleic acid structures, the vaccine against human smallpox provides cross-protection against Mpox. Individuals vaccinated against smallpox before 1980, when vaccination ceased following eradication of the disease, tend to experience milder or asymptomatic infections. However, this residual immunity is limited to older age groups [8]. The lower virulence and lethality observed during the 2022 global outbreak were not attributable to vaccination but rather to the Clade IIb variant, which causes milder forms of disease characterized by localized lesions (genital, anal, or oral), fewer lesions overall, minimal fever, and a self-limited clinical course. In contrast, the more aggressive Clade I variant, which remains predominant in Central Africa, is associated with high fever, extensive lymphadenopathy, and widespread skin lesions [9].

Mpox remains a rare condition during pregnancy, with the majority of available data derived from isolated case reports, small case series, and historical observations from endemic regions in Central and West Africa. During the 2022–2023 global outbreak, only a limited number of pregnancy-associated cases were reported, precluding robust estimates of incidence or risk. Importantly, the available epidemiological data do not allow precise quantification of maternal or fetal risk, as reporting bias and underdiagnosis are likely, particularly in low-resource settings. Consequently, current epidemiological knowledge should be interpreted with caution, emphasizing trends rather than definitive risk estimates [10,11].

This review aims to provide a comprehensive overview of the Monkeypox virus, describing its epidemiology, clinical presentation, transmission routes, preventive measures, and therapeutic approaches, with emphasis on the challenges and particularities observed in pregnant and postpartum women and infants.

## 2. General Aspects of the Monkeypox Virus

The Monkeypox virus is a zoonotic pathogen belonging to the family Poxviridae and the genus Orthopoxvirus, which also includes the Variola virus (smallpox virus), the Vaccinia virus, and the Cowpox virus [12,13]. It is a large, double-stranded deoxyribonucleic acid virus with a complex morphology and a characteristic brick-shaped structure surrounded by multiple lipid membranes. Its structural architecture and extensive protein repertoire confer remarkable environmental stability, surpassing that of many ribonucleic acid viruses [13,14].

Unlike most deoxyribonucleic acid viruses, the Monkeypox virus can be replicated entirely within the cytoplasm of the host cell. This is possible because its genome encodes its own transcription and replication machinery, allowing relative independence from the host cell nucleus and complete assembly of viral particles in the cytoplasm [14,15]. This characteristic contributes to its broad cellular tropism and potential to cause systemic manifestations in susceptible hosts.

The virus was first identified in 1958 in laboratory monkeys in Denmark, which gave rise to its common name, “monkeypox.” The first human case was documented in 1970 in the Democratic Republic of the Congo during surveillance activities for smallpox, shortly after its eradication [15,16]. Since then, Monkeypox virus has remained endemic in regions of Central and West Africa, with zoonotic cycles involving several wild mammals, particularly rodents such as squirrels of the genus *Funisciurus*, which are considered the primary natural reservoirs [17,18]. Non-human primates and humans are incidental hosts.

In 2022, the World Health Organization (WHO) recommended renaming the disease to “mpox” to reduce stigma and prevent discriminatory or misleading associations with primates or specific geographic regions. The name “monkeypox” had increasingly been linked to racist or xenophobic narratives, and it also inaccurately implied that monkeys were the primary reservoirs, when, in fact, rodents are more likely to serve as the main hosts. The transition to “mpox” sought to promote neutral, non-stigmatizing language in global public health communication while maintaining clarity for clinicians and researchers.

Transmission occurs through direct contact with bodily fluids, crusts, or skin lesions of infected individuals; through respiratory droplets during close and prolonged contact; and through contaminated fomites, such as bedding, clothing, and surfaces [19]. Beginning in 2022, a substantial change in epidemiological patterns occurred, with sustained human-to-human transmission in multiple non-endemic countries. This global outbreak underscored the potential for viral dissemination through interpersonal contact within social networks and intimate environments, prompting the WHO to declare a Public Health Emergency of International Concern (PHEIC) in July 2022 [20,21].

From a genetic standpoint, Monkeypox virus is classified into two major clades (Table 1):

- Clade I (Congo Basin/Central Africa)—associated with greater virulence and lethality;
- Clade II (West Africa)—generally responsible for milder clinical forms, including the 2022 global outbreak [22].

**Table 1:** Key differences between clade I and clade II of the Monkeypox virus.

Characteristic	Clade I (Congo Basin/Central Africa)	Clade II (West Africa, including Clade IIb)
Virulence	Higher virulence; associated with more severe systemic disease	Lower virulence; typically causes milder clinical presentations
Case-Fatality Rate	Historically 8–10%	Typically <1% in recent outbreaks
Clinical Features	High fever, extensive lymphadenopathy, widespread lesions	Frequently localized lesions (genital, anal, oral), fewer systemic symptoms
Transmission Dynamics	More efficient human-to-human transmission	Sustained human-to-human transmission documented mainly since 2022
Geographic Distribution	Endemic in Central Africa (e.g., DRC, CAR)	Historically in West Africa; since 2022, global dissemination
Genomic Characteristics	More genetically conserved; associated with higher pathogenicity	Displays ongoing microevolution and emergence of new lineages (e.g., IIb)
Predominant Populations Affected	Rural populations with animal exposure	Urban adults with close-contact or sexual transmission networks
Role in 2022 Outbreak	Not involved	Major lineage responsible (Clade IIb)

Recent studies indicate ongoing genomic microevolution and viral diversification, with the emergence of new lineages, reinforcing the need for continuous genomic surveillance [23]. The decline in population-level immunity following smallpox eradication in 1980 has contributed to Monkeypox virus reemergence in recent decades, as vaccination against the Variola virus provided partial cross-protection against other Orthopoxviruses [14].

Although the disease is typically self-limited, severe forms may occur in young children, immunosuppressed individuals, and pregnant women [24,25]. Case-fatality rates depend on viral clade, healthcare quality, and associated comorbidities.

From a public health perspective, the global response between 2022 and 2024 included intensified laboratory surveillance (polymerase chain reaction testing for viral deoxyribonucleic acid), contact tracing, case isolation, targeted vaccination, and use of antivirals such as tecovirimat in specific clinical contexts [26]. Maintaining genomic and epidemiological surveillance remains essential for understanding viral adaptation, the clinical impact of new variants, and the long-term effectiveness of control strategies.

According to the 2023 RCOG guideline, infections caused by Clade II mpox (the strain responsible for the 2022–2023 global outbreak) are no longer classified as High Consequence Infectious Diseases (HCID). In contrast, Clade I infections, endemic in Central Africa, remain HCID due to their higher virulence, greater case-fatality rate, and increased risk of severe maternal outcomes. This distinction has immediate clinical implications for pregnant patients, as Clade II disease typically presents with milder systemic manifestations and lower mortality but still warrants enhanced obstetric surveillance [14].

## 3. Clinical Presentation

Infection caused by the Monkeypox virus is characterized by a broad range of systemic and cutaneous manifestations (Table 2). Historically, clinical features were mainly derived from outbreaks caused by Clade I, predominant in Central Africa, which typically exhibited a centrifugal rash, high rates of lymphadenopathy, and a more severe systemic illness, particularly among children. However, since the global spread of Clade II beginning in 2022, a distinct clinical profile has emerged, marked by milder systemic symptoms, frequent localized anogenital lesions, and atypical or asynchronous cutaneous manifestations.

Cutaneous eruptions occur in approximately 96% of cases and are frequently followed by influenza-like and gastrointestinal symptoms, including fever, lymphadenopathy, and secondary skin infections. Symptoms may emerge abruptly and progress gradually, with an average duration of two to four weeks, although the severity and clinical course vary according to the patient’s immune status and individual conditions [27].

The incubation period ranges from seven to twenty-one days, and nearly all patients report nonspecific early symptoms such as fever, headache, myalgia, and lymphadenopathy, followed by the appearance of cutaneous lesions [27]. Fever typically precedes the dermatological eruptions, which tend to develop approximately five days after fever onset. The lesions progress through successive stages, beginning as macules and evolving into papules, vesicles, pustules, and ultimately crusts, which may leave residual scarring once resolved [28].

In Clade I outbreaks, eruptions classically began on the face, spreading centrifugally to the trunk and extremities, including the palms and soles. In contrast, Clade II infection frequently presents with initial mucocutaneous lesions in the genital, perianal, or oral regions, sometimes without prodromal fever or with minimal systemic symptoms. Lesions may be fewer in number, asynchronous, and occasionally associated with rectal pain, proctitis, and localized lymphadenopathy, features rarely emphasized in Clade I infections.

Although clinical presentation may resemble that of human smallpox, Mpox infection tends to follow a milder course. Nonetheless, severe or fatal outcomes may still occur—especially in children and young adults affected by secondary complications such as bacterial infections, encephalitis, sepsis, and in pregnant women [28]. The shift in clinical patterns associated with Clade II underscores the need for updated diagnostic suspicion and tailored public-health strategies.

## 4. Transmission

The definitive reservoir of the Monkeypox virus has not yet been conclusively identified, although numerous scientific investigations have sought to clarify this issue. The first documented isolation of the virus in a wild animal occurred in an infected squirrel, and since then, additional rodent species have been recognized as potential reservoirs, expanding the range of susceptible hosts. Furthermore, in 2012, the virus was isolated from a wild sooty mangabey and subsequently in populations of chimpanzees (*Pan troglodytes verus*) in Côte d’Ivoire, reinforcing the hypothesis that several wild mammals participate in the natural maintenance cycle of Monkeypox [27,28,29].

Evidence regarding vertical transmission of mpox remains limited. Molecular confirmation of in utero infection, such as PCR-positive placental or fetal tissue, has been documented only in isolated cases. Most descriptions suggesting vertical transmission derive from small observational series or historical cohorts, often without standardized diagnostic confirmation. Consequently, while vertical transmission appears biologically plausible, its frequency and clinical significance remain uncertain [30].

## 5. Prevention

Preventing infection caused by the Monkeypox virus represents a significant challenge, particularly in endemic regions where cultural, social, and economic factors hinder the implementation of effective control measures. The primary preventive strategy is to reduce contact between humans and potentially infected animals, especially rodents and primates, which are considered the main reservoirs of the virus. In this context, the handling and consumption of meat from wild animals (“bushmeat”) constitute an important route of exposure and should be strongly discouraged. However, in areas marked by socioeconomic vulnerability, the consumption of such meat is often a primary source of protein, making enforcement and prohibition difficult for public health authorities [28].

Health education, therefore, plays a fundamental role in disease prevention. Public awareness campaigns are essential to inform communities about the use of protective equipment such as gloves, masks, and appropriate clothing when handling animals that may be contaminated [28]. Additionally, close contact with individuals presenting cutaneous eruptions compatible with Mpox should be avoided, along with the sharing of personal items such as utensils, drinking vessels, and clothing. Simple hygiene measures, including regular handwashing before meals and after using the restroom, are also indispensable for reducing viral transmission [29].

When combined with effective public policies and large-scale awareness initiatives, these measures can significantly contribute to controlling the spread of Mpox, particularly in communities at heightened risk of exposure [28,29].

## 6. Mpox in the Obstetric Population

The scientific literature on Monkeypox virus infection during pregnancy remains notably limited, resulting in a substantial scarcity of data for this specific population. Occurrence during gestation is considered rare, which contributes to the existing knowledge gap regarding its clinical manifestations and potential maternal–fetal repercussions [30,31].

According to the most recent WHO and national surveillance data, only small international cohorts of pregnant or recently pregnant individuals with mpox have been reported. The 2022–2023 WHO global trends report identified 55 such cases worldwide, of whom 12 required hospitalization, but none required intensive care or resulted in maternal deaths. In Brazil, epidemiological data documented 22 pregnant women with confirmed or suspected mpox, including two who required hospital admission. In the United States, the CDC reported 17 confirmed or probable cases in pregnant or recently pregnant individuals. Although these numbers remain limited, the available data are generally reassuring and suggest that, in most instances, the clinical course of Clade II mpox during pregnancy is mild [14].

Given this uncertainty, careful obstetric surveillance is essential, particularly in symptomatic pregnant individuals or those with confirmed Mpox infection, considering the possibility of vertical transmission. Ultrasonographic monitoring plays a central role in this context, as certain findings may suggest fetal compromise, including hepatomegaly, ascites, fetal hydrops, placental calcifications, and intrauterine growth restriction [31].

Clinical diagnosis in pregnant individuals, however, may present substantial challenges. Fever, a common symptom of Monkeypox virus infection, may also result from alternative etiologies such as intra-amniotic infection; therefore, exclusion of other infectious and non-infectious causes is indispensable. Similarly, cutaneous manifestations require meticulous evaluation, as they may be mistaken for pregnancy-specific dermatoses, such as pruritic urticarial papules and plaques of pregnancy, or for more prevalent infections, including varicella–zoster virus or sexually transmitted infections. Thus, a detailed clinical examination, together with consideration of the epidemiological context, should guide the indication for diagnostic testing for Mpox [32].

Adverse pregnancy outcomes, including miscarriage, stillbirth, and preterm birth, have been described in association with maternal mpox infection. However, these outcomes are primarily reported in small case series and historical cohorts from endemic regions. The absence of control groups, potential reporting bias, and frequent coexistence of confounding factors limit causal inference.

Given that current evidence is largely derived from isolated case reports and small observational series, we summarized all published cases of mpox infection during pregnancy in Table 3, highlighting maternal characteristics, timing of infection, clinical presentation, and reported maternal–fetal outcomes.

## 7. Diagnosis

Detection of the Monkeypox virus is an essential diagnostic requirement, performed according to standardized technical protocols in adequately equipped laboratories. Confirmation of infection relies primarily on amplification of viral deoxyribonucleic acid through polymerase chain reaction testing [38].

Two main diagnostic algorithms are currently used. The first involves initial detection of generic Orthopoxviruses, followed by specific confirmation of Monkeypox virus through polymerase chain reaction or sequencing. The second, now recommended, performs direct detection of Monkeypox virus with subsequent differentiation between clades I and II. When an Orthopoxvirus assay yields a positive result, specific confirmation of Monkeypox virus becomes mandatory in regions where multiple Orthopoxviruses circulate [38].

Laboratory confirmation requires integration of molecular findings with clinical and epidemiological information. Sequencing of viral genetic material is a valuable complementary tool, allowing precise identification of the agent and improving understanding of the virus’s origin and evolutionary characteristics. Sequencing may be performed through Sanger methodology or next-generation sequencing. It is recommended that sequencing be conducted on the broadest possible sample of positive specimens from different patients and locations [38].

False-negative results may arise from inadequate sample quality, incorrect assay configuration, or failures during transport. When the clinical evaluation suggests Monkeypox virus infection despite a negative polymerase chain reaction result, serological testing may assist in further epidemiological investigation [7]. Cases with laboratory confirmation must be immediately reported to WHO and to the relevant public health authorities [38].

The most appropriate samples for laboratory investigation of Monkeypox virus are obtained from skin lesions, particularly from vesicular fluid or crust material. These samples must be preserved in sterile containers without viral additives and maintained at low temperatures. When clinically indicated, oral or nasal samples may also be collected [28,38].

For serological evaluation based on antibody detection, blood samples should be collected at two distinct time points: during the initial phase of the disease and during recovery. The objective is to detect immunoglobulin M (early response) within the first five days after symptom onset, or immunoglobulin G (late response) after approximately one week of evolution [28].

## 8. Treatment of Mpox, Including Supportive Care, Antiviral Therapy, and Considerations During Pregnancy and Lactation

Management of mpox infection is primarily supportive in most cases, as the disease is generally self-limited. Standard treatment includes adequate analgesia, antipyretics, hydration, correction of electrolyte imbalances, and nutritional support. Local care of cutaneous and mucosal lesions is essential to reduce pain, prevent secondary bacterial infection, and promote healing. This includes gentle cleansing, topical antiseptics when indicated, and appropriate wound care. Systemic antibiotics should be reserved for documented or strongly suspected secondary bacterial infections, such as cellulitis, pneumonia, or sepsis, and are not routinely indicated [39,40,41]. Patients should remain in isolation until complete resolution of lesions with re-epithelialization to minimize viral transmission [40,41].

Hospitalization is recommended for individuals with severe disease, extensive mucocutaneous involvement, complications such as dehydration or bacterial superinfection, significant pain requiring parenteral analgesia, or involvement of critical anatomical sites (e.g., ocular, neurologic, or respiratory disease). Immunocompromised patients, including those with advanced human immunodeficiency virus infection, may require closer monitoring due to a higher risk of severe or prolonged disease [35,42].

Pharmacological antiviral therapy may be considered in selected patients with severe mpox, those at high risk for complications, or individuals with significant immunosuppression. Currently available antiviral options include tecovirimat, cidofovir, brincidofovir, and vaccinia immune globulin, although evidence supporting their use is largely derived from animal models, in vitro studies, and limited observational human data. Under the World Health Organization’s Monitored Emergency Use of Unregistered and Investigational Interventions (MEURI) framework, tecovirimat has been authorized for emergency use during the current outbreak, allowing controlled access while facilitating systematic collection of safety and outcome data [42].

Tecovirimat inhibits the viral VP37 (p37) protein, which is required for the formation of extracellular enveloped virions, thereby limiting viral dissemination between host cells [43,44]. Preclinical studies have demonstrated significant activity against Orthopoxviruses, including Monkeypox virus, with reductions in viral load and mortality in animal models. Observational human data suggest potential clinical benefit, particularly in severe disease, although randomized clinical trials have shown mixed results regarding time to lesion resolution [21,43,44]. Tecovirimat is generally well tolerated, with reported adverse effects including gastrointestinal symptoms, headache, and transient elevations in liver enzymes. Its use is currently recommended primarily for severe disease, high-risk populations, or within regulated clinical protocols, as inappropriate widespread use may contribute to the emergence of antiviral resistance [42].

Cidofovir is a nucleotide analog that inhibits viral DNA polymerase after intracellular phosphorylation, thereby impairing viral replication [45,46,47]. Although in vitro and animal studies suggest antiviral activity against Monkeypox virus, clinical experience in humans is limited. Its use is constrained by significant nephrotoxicity, requiring prehydration, coadministration of probenecid, and close renal monitoring. Brincidofovir, an oral lipid-conjugated prodrug of cidofovir, was developed to improve bioavailability and reduce renal toxicity. Emergency use authorization permits its use in selected severe cases, particularly in immunocompromised individuals or when other therapeutic options are unavailable or contraindicated [21]. Vaccinia immune globulin may be considered in severe cases or in patients with impaired humoral immunity, although data regarding its effectiveness specifically for mpox remain limited [40,48].

Treatment considerations during pregnancy and lactation require particular caution. Pregnant individuals with mpox may be at increased risk for maternal complications and adverse fetal outcomes and therefore warrant close clinical monitoring and a low threshold for hospitalization [40,41,47]. Supportive care principles remain the cornerstone of management and should be optimized to maintain maternal stability and fetal well-being. When antiviral therapy is indicated during pregnancy, tecovirimat is currently regarded as the preferred option. Available preclinical animal data have not demonstrated teratogenicity or significant maternal–fetal toxicity, supporting its cautious use when the potential benefits outweigh theoretical risks [49]. In contrast, cidofovir and brincidofovir are contraindicated during pregnancy due to teratogenic and embryotoxic effects observed in animal studies, precluding their use in pregnant individuals [46,47].

Decisions regarding antiviral therapy during pregnancy and lactation should be individualized, taking into account disease severity, gestational age, maternal comorbidities, and fetal status, and ideally should involve a multidisciplinary team including obstetricians, infectious disease specialists, and neonatologists. Overall, optimal management of mpox relies on a combination of supportive care, judicious use of antivirals in selected cases, and careful risk–benefit assessment, particularly in vulnerable populations such as pregnant and lactating individuals.

## 9. Vaccination Against Mpox: General Principles, Adult Use, and Considerations During Pregnancy

The global emergence of mpox required the rapid adoption of prevention and control strategies, among which vaccination represents a cornerstone for limiting viral dissemination and reducing severe disease [1]. Vaccination against mpox is based on cross-protection among Orthopoxvirus species, given the close genetic relationship between the Monkeypox virus and the Variola virus [50]. Historical observational studies and recent epidemiological analyses have demonstrated that smallpox vaccination confers up to 85% protection against mpox infection [51,52]. Following the eradication of smallpox in 1980 and the subsequent discontinuation of mass vaccination programs, population immunity against Orthopoxviruses progressively declined, contributing to increased susceptibility during the 2022 global outbreak [4].

In this context, international health authorities, including the World Health Organization and the United States Centers for Disease Control and Prevention, recommended the preferential use of third-generation smallpox vaccines with improved safety profiles [51,52,53,54]. Currently, the Modified Vaccinia Ankara–Bavarian Nordic (MVA-BN) vaccine—commercialized as Jynneos, Imvanex, or Imvamune—is the main formulation approved by international regulatory agencies for mpox prevention [55,56,57,58]. MVA-BN is a live, attenuated, non-replicating Orthopoxvirus vaccine developed through genetic modification, resulting in a substantially safer profile compared with earlier replicating vaccines such as ACAM2000 and Dryvax [55,56,59]. ACAM2000, a replicating Vaccinia-based vaccine, remains effective but is associated with higher rates of adverse events, including myocarditis and extensive cutaneous reactions, and is therefore reserved for exceptional circumstances such as contraindications to MVA-BN or public health emergencies [54,57].

The standard MVA-BN vaccination regimen consists of two subcutaneous doses administered 28 days apart, with effective immunological protection achieved approximately two weeks after the second dose [55]. Its mechanism of action involves activation of both cellular and humoral immune responses against Orthopoxvirus antigens. Cellular immunity is characterized by induction of CD4^+^ and CD8^+^ T-cell responses, leading to cytotoxic activity against infected cells, while humoral immunity results from B-cell activation and antibody production directed against orthopoxviral proteins [3]. Although natural mpox infection generally induces stronger antibody responses, MVA-BN vaccination elicits more robust T-cell responses, supporting a faster and more effective immune reaction upon subsequent exposure [59,60].

Vaccination strategies include both pre-exposure prophylaxis (PrEP) and post-exposure prophylaxis (PEP). Pre-exposure prophylaxis is recommended for individuals at increased risk of infection, including healthcare professionals handling biological materials and populations with higher exposure risk, such as people living with human immunodeficiency virus/acquired immunodeficiency syndrome [55]. Post-exposure prophylaxis is indicated for individuals with close contact with suspected or confirmed mpox cases and should preferably be administered before or shortly after exposure to prevent infection or attenuate disease severity [50,52,56]. Real-world data suggest that MVA-BN vaccination provides approximately 76% effectiveness after the first dose and 82% after completion of the two-dose schedule, with a reported 67% reduction in hospitalizations among vaccinated individuals, reinforcing its role in preventing severe outcomes [19,49].

In Brazil, mpox vaccination was implemented on 13 March 2023, prioritizing individuals at increased vulnerability or exposure risk. Between 2022 and 2023, 9596 mpox cases were recorded nationwide, and approximately 49,000 vaccine doses were distributed, of which 14,395 (31%) were administered [55]. Pharmacovigilance surveillance identified 65 events supposedly attributable to vaccination or immunization; all were classified as non-serious, with the most common manifestations including injection-site pain and swelling, headache, fever, and myalgia. No deaths or serious adverse events were reported. Nevertheless, the relatively limited number of administered doses may restrict detection of rare adverse events, underscoring the importance of ongoing surveillance and strengthened monitoring systems [55].

Vaccination during pregnancy warrants particular consideration, as pregnant individuals may be more vulnerable to severe maternal disease and adverse fetal outcomes [49,56]. MVA-BN’s non-replicating nature supports its preferential use in pregnant and lactating individuals at risk of infection, in contrast to earlier replicating vaccines such as ACAM2000 or LC16m8, which are contraindicated during pregnancy due to safety concerns [49,56]. Although clinical evidence remains limited, available preclinical and observational data suggest a favorable safety profile for MVA-BN in pregnancy. Animal studies have not demonstrated teratogenicity, fetal toxicity, or maternal–fetal adverse outcomes attributable to vaccination [49,56]. However, no randomized clinical trials specifically evaluating vaccine safety or effectiveness in pregnant populations have been completed to date, and current recommendations rely on indirect evidence and expert consensus [48].

In the context of mpox infection during pregnancy, antiviral therapy may also be considered. Tecovirimat is currently regarded as the safest antiviral option for pregnant individuals when treatment is indicated, whereas antivirals such as cidofovir and brincidofovir are contraindicated due to teratogenic and embryotoxic effects observed in animal studies [49]. Vaccination, therefore, remains a central component of mpox prevention strategies, particularly in high-risk settings, and should be integrated with surveillance, contact tracing, and public health interventions to mitigate maternal and fetal risks [58,59].

## 10. Post-Exposure Measures

Individuals exposed to the virus may continue daily activities if no signs or symptoms develop. People with skin-to-skin contact, droplet exposure, contact with contaminated surfaces, or contact with infected animals should be monitored for 21 days. Monitoring includes a complete examination of the skin and mucous membranes, including the genital area, to assess for rash, fever, or lymphadenopathy. If a skin lesion appears, the individual should remain isolated until test results are negative. If no rash is present but other symptoms occur, a five-day isolation period is required, even beyond the initial 21 days. Any new symptom resets this five-day isolation requirement. If no further symptoms arise after the initial five days, isolation can be discontinued [61].

Isolation recommendations include avoiding visitors, maintaining physical distance from others and pets, abstaining from sexual activities involving direct contact, and not sharing potentially contaminated objects. Frequently touched surfaces should be routinely cleaned and disinfected. Surgical masks should be used during interaction with others. Patients with lesions should avoid contact lenses and refrain from shaving affected areas. Ideally, a separate bathroom should be used; otherwise, surfaces must be disinfected after each use. Gloves should be worn when handling dressings or cleaning contaminated areas. Isolation should continue until all crusts have detached and new skin has formed [62].

Post-exposure management is guided by exposure-risk classification, which determines whether prophylaxis is needed. Brief or superficial interactions rarely result in transmission; risk increases with physical proximity and duration. Public health authorities should classify risk in situations where individual exposure cannot be determined [61].

High-risk exposure includes direct contact between broken skin, mucous membranes, bodily fluids, or materials contaminated with lesions from a person with Mpox. These individuals require monitoring and post-exposure prophylaxis unless they have already completed the full vaccination schedule [61].

Intermediate-risk exposure includes contact between intact skin and the lesions or bodily fluids of an infected person or visibly contaminated materials, as well as being within 2 m of an infected individual with laryngeal disease or respiratory symptoms. Monitoring is required, and post-exposure vaccination may be considered after risk–benefit assessment; additional vaccine doses are not recommended for fully vaccinated individuals [61].

Minimal or uncertain risk includes entering the living space of an infected person or contact between intact skin or clothing of both individuals. Monitoring is at the discretion of public health authorities, and post-exposure prophylaxis is not recommended [61]. In the absence of any defined exposure, no monitoring or post-exposure prophylaxis is required. Risk classification may be reassessed by healthcare professionals as needed [61].

Post-exposure prophylaxis aims to reduce or prevent infection. The recommended vaccine is the third-generation non-replicating MVA-BN (Imvanex, Jynneos, or Imvamune), ideally administered within four days of exposure, with an extended window of up to 14 days if the individual remains asymptomatic. The schedule involves two doses 28 days apart. Replicating vaccines such as Dryvax and ACAM2000 must not be used for prophylaxis [63,64].

For obstetric patients, non-pharmacological prevention is the priority. Vaccination may be considered only in high-risk situations and when benefits outweigh risks. ACAM2000 is strictly contraindicated for post-exposure prophylaxis in pregnancy [31].

For individuals managed with non-pharmacological measures, additional precautions are essential. These include frequent hand hygiene, use of appropriate personal protective equipment by healthcare workers, and disinfection of contaminated surfaces. The virus can persist for weeks on porous materials, highlighting the importance of environmental control with sodium hypochlorite, alcohol-based solutions, or hospital-grade detergents. Textiles should be washed with hot water and soap, and contaminated materials must be disposed of safely [65].

## 11. Population, Ethics, and Risk of Discrimination

Severe forms of Mpox infection predominantly affect children, pregnant individuals, and immunosuppressed patients, especially those living with uncontrolled Human Immunodeficiency Virus (HIV). Overall, Mpox has disproportionately impacted men who have sex with men, individuals living with HIV, and racial and ethnic minorities, particularly in non-endemic regions. Recent epidemiological data indicate that approximately ninety-five percent of all reported cases have occurred among men who have sex with men, frequently with high rates of coinfection with HIV and other sexually transmitted infections [15,17,19,66,67,68,69].

From a medical and ethical standpoint, the promotion of health necessarily involves identifying populations at increased risk for any infection in order to guide adequate public health interventions, including targeted vaccination and diagnostic strategies. However, defining such groups may lead to stigmatization and discrimination, particularly when epidemiological trends indicate a higher concentration of cases within specific communities. In this context, the American College of Occupational and Environmental Medicine emphasizes that, although recognizing risk groups is essential for effective transmission control, preventing stigma is equally critical, as discriminatory narratives can intensify fear, perpetuate inequalities, and hinder access to healthcare. Therefore, clear, evidence-based, and socially sensitive communication strategies are recommended to ensure equitable access to prevention and treatment without reinforcing prejudice. Consequently, the current literature underscores that combating discrimination is an integral component of the response to monkeypox. Reducing stigma and promoting equity must occur in parallel with focused and effective epidemiological surveillance [66,67].

## 12. Conclusions

Mpox infection during pregnancy represents a challenging clinical scenario characterized by limited evidence and substantial uncertainty. Although cases of severe maternal disease and adverse fetal outcomes have been reported, the current literature does not allow precise estimation of absolute risks or definitive causal inferences. Consequently, clinical management should prioritize individualized risk assessment, adherence to evolving guidelines, and cautious interpretation of the available data.

Importantly, the interpretation of mpox infection in pregnancy is constrained by significant methodological limitations. Most published evidence is derived from case reports, small case series, and historical cohorts, frequently lacking control groups, standardized diagnostic criteria, and systematic follow-up. Reporting bias is likely, and data from endemic regions often reflect healthcare settings with limited diagnostic capacity and prenatal care resources. As a result, many associations described in the literature should be regarded as suggestive rather than definitive.

Prospective studies and pregnancy-specific surveillance registries are urgently needed to better define maternal, fetal, and neonatal risks and to support evidence-based clinical decision-making in this vulnerable population.

## Figures and Tables

**Table 2:** Maternal, fetal, and neonatal findings reported in Mpox infection during pregnancy.

Domain	Reported Findings	Comments/Evidence
Maternal Symptoms	Fever, rash, lymphadenopathy, mucocutaneous lesions	Similar to the general adult population; severity may be higher in pregnancy
Maternal Complications	Pneumonia, sepsis, hospitalization in severe cases	Limited data; risk appears increased in immunocompromised or late gestation
Ultrasound Findings Suggesting Fetal Compromise	Hepatomegaly, ascites, hydrops fetalis, placental calcifications, intrauterine growth restriction	Based on case reports and small series; no large cohort data available
Risk of Vertical Transmission	Possible transplacental transmission; PCR-positive fetal and placental tissues documented	Evidence suggests risk across all trimesters, though overall frequency is unknown
Fetal Outcomes	Miscarriage, stillbirth, intrauterine fetal demise	Systematic reviews report fetal loss in approximately half of documented cases
Neonatal Outcomes	Neonatal rash, respiratory distress, sepsis-like presentations	Transmission may occur in utero, intrapartum, or immediately postpartum
Differential Diagnosis Challenges	Cutaneous lesions may resemble pregnancy dermatoses (e.g., PUPPP) or varicella–zoster	Requires careful clinical assessment and molecular confirmation
Monitoring Recommendations	Serial ultrasonography, fetal growth assessment, biophysical profile in moderate–severe cases	Suggested by WHO and CDC given limited evidence base

**Table 3:** Reported cases of Mpox infection during pregnancy and associated maternal–fetal outcomes.

Reference	Country	Age	Gestational Age at Infection	Clinical Presentation	Coinfection	Fetal Complication	Gestational Age at Birth
Mbala et al. [33]	Democratic Republic of the Congo	20	6	Vesicular rash and/or enanthems in the oral cavity and a history of fever	NR	Miscarriage	Not applicable
		25	6–7		NR	Miscarriage	Not applicable
		29	14		NR	Livre birth	Term
		22	18		Malaria	Fetal death	Not applicable
Yinka-Ogunleye et al. [34]	Nigeria	NR	NR	NR	NR	Fetal death (26 weeks)	Not applicable
Ogoina et al. [35]	Nigeria	NR	1st–2nd trimester	PROM	NR	Miscarriage (16 weeks)	Not applicable
Oakley et al. [36]	United States	19	24	Vaginitis	HIV negative	No	37 weeks
		23	36	Asymptomatic	HIV negative	No	39 weeks
García-Hernández et al. [37]	Spain	24	Since conception	Asthenia, localized lymphadenopathy, and burning rash in the genital region	Chlamydia trachomatis (PCR) was found 27 days after mpox confirmation	No	38 weeks

NR: Not reported. PROM: Premature rupture of membranes.

## Data Availability

The data presented in this study are available on request from the corresponding author.

## References

[1] Elsayed S., Bondy L., Hanage W.P. (2022). Monkeypox Virus Infections in Humans. Clin. Microbiol. Rev..

[2] Ministério da Saúde do Brasil (2022). O Que é Monkeypox (Mpox).

[3] Ministério da Saúde do Brasil (2023). Mpox (Monkeypox). https://bvsms.saude.gov.br/mpox/.

[4] World Health Organization (WHO) (2022). Multi-Country Outbreak of Monkeypox: External Situation Report #5–2022.

[5] Centers for Disease Control and Prevention (CDC) (2023). About Mpox (Monkeypox).

[6] Ministério da Saúde do Brasil (2023). Protocolo Clínico e Diretrizes Terapêuticas da Infecção Pelo Vírus Monkeypox (Mpox).

[7] Organização Pan-Americana da Saúde (OPAS) (2022). Relatório Semanal da Situação Sobre a Resposta ao Surto da Varíola dos Macacos em Vários Países—Região das Américas 22 de Julho de 2022.

[8] Pérez-García J.A., Dans-Vilan L., Montero-Morales L., Culebras E., Fernández-Castelao S., Miguel-Benito Á., Puerta-López T., García-Campero A.D.I. (2025). First case of Mpox clade Ib transmitted from human-to-human outside the African continent and not linked to travel. Int. J. Infect. Dis..

[9] World Health Organization (WHO) (2023). Monkeypox: Key Facts.

[10] Camones-Huerta J., Cordero-Campos A., Cuya-Sahua A., Guzmán-Carrasco S., Condori-Quispe L., Chin-Wu K., Quispe-Villegas G., Flores-Noriega M. (2025). Monkeypox infection in pregnancy, maternal and fetal outcomes: A systematic review. J. Infect. Dev. Ctries..

[11] Centers for Disease Control and Prevention (CDC) (2025). Monkeypox Clinical Care and Treatment During Pregnancy.

[12] World Health Organization (WHO) (2024). Mpox (Monkeypox)—Fact Sheet.

[13] Alakunle E.F., Okeke M.I. (2022). Monkeypox virus: A neglected zoonotic pathogen spreads globally. Nat. Rev. Microbiol..

[14] Alakunle E., Moens U., Nchinda G., Okeke M.I. (2020). Monkeypox Virus in Nigeria: Infection Biology, Epidemiology, and Evolution. Viruses.

[15] Gessain A., Nakoune E., Yazdanpanah Y. (2022). Monkeypox. N. Engl. J. Med..

[16] The Lancet Infectious Diseases (2022). Monkeypox: A neglected old foe. Lancet Infect. Dis..

[17] Mitjà O., Ogoina D., Titanji B.K., Galvan C., Muyembe J.J., Marks M., Orkin C.M. (2023). Monkeypox. Lancet.

[18] Isidro J., Borges V., Pinto M., Sobral D., Santos J.D., Nunes A., Mixão V., Ferreira R., Santos D., Duarte S. (2022). Phylogenomic characterization and signs of microevolution in the 2022 multi-country outbreak of monkeypox virus. Nat Med..

[19] Thornhill J.P., Antinori A., Orkin C.M. (2022). Monkeypox Virus Infection across 16 Countries—April–June 2022. N. Engl. J. Med..

[20] World Health Organization (2022). WHO Director-General Declares the Ongoing Monkeypox Outbreak a Public Health Emergency of International Concern.

[21] Centers for Disease Control and Prevention (2024). Clinical Care Guidance: Monkeypox.

[22] Johns Hopkins University (2024). Mpox Virus: Clade I and Clade II.

[23] Hirani R., Noruzi K., Iqbal A., Hussaini A.S., Khan R.A., Harutyunyan A., Etienne M., Tiwari R.K. (2023). A review of the past, present, and future of the Monkeypox virus. Vaccines.

[24] Ahmadi S., Amirzadeh M., Ahmadi M., Soleiman-Meigooni S. (2024). From Outbreaks to Artificial Intelligence: A Comprehensive Review of Monkeypox Virus Epidemiology, Diagnosis, Treatment, Vaccination, and Deep Learning Applications. J. Trop. Med..

[25] World Health Organization (2025). Clinical Management and Infection Prevention and Control for Mpox: Living Guideline.

[26] Centers for Disease Control and Prevention (2024). Tecovirimat (TPOXX): Treatment and Recommendations for Mpox.

[27] Karagoz A., Tombuloglu H., Alsaeed M., Tombuloglu G., AlRubaish A.A., Mahmoud A., Smajlović S., Ćordić S., Rabaan A.A., Alsuhaimi E. (2023). Monkeypox (mpox) virus: Classification, origin, transmission, genome organization, antiviral drugs, and molecular diagnosis. J. Infect. Public. Health.

[28] Petersen E., Kantele A., Koopmans M., Asogun D., Yinka-Ogunleye A., Ihekweazu C., Zumla A. (2019). Human Monkeypox: Epidemiologic and Clinical Characteristics, Diagnosis, and Prevention. Infect. Dis. Clin. N. Am..

[29] Huang Y., Mu L., Wang W. (2022). Monkeypox: Epidemiology, pathogenesis, treatment and prevention. Signal Transduct Target Ther..

[30] Cuérel A., Favre G., Vouga M., Pomar L. (2022). Monkeypox and Pregnancy: Latest Updates. Viruses.

[31] Dashraath P., Nielsen-Saines K., Rimoin A., Mattar C.N.Z., Panchaud A., Baud D. (2022). Monkeypox in pregnancy: Virology, clinical presentation, and obstetric management. Am. J. Obstet. Gynecol..

[32] Abu-Azzam O., Abu-Jeyyab M., Daradkeh M., Eldeen S.Z., Zuaiter S., Al-Mseadeen M., Sindiani A., Alshdaifat E. (2024). Monkeypox infection and pregnancy in lower and middle-income countries: Precautions & recommendations. Rev. Bras. Ginecol. Obstet..

[33] Mbala P.K., Huggins J.W., Riu-Rovira T., Ahuka S.M., Mulembakani P., Rimoin A.W., Martin J.W., Muyembe J.T. (2017). Maternal and Fetal Outcomes Among Pregnant Women With Human Monkeypox Infection in the Democratic Republic of Congo. J. Infect. Dis..

[34] Yinka-Ogunleye A., Aruna O., Dalhat M., Ogoina D., McCollum A., Disu Y., Mamadu I., Akinpelu A., Ahmad A., Burga J. (2019). Outbreak of human monkeypox in Nigeria in 2017-18: A clinical and epidemiological report. Lancet Infect. Dis..

[35] Ogoina D., Iroezindu M., James H.I., Oladokun R., Yinka-Ogunleye A., Wakama P., Otike-Odibi B., Usman L.M., Obazee E., Aruna O. (2020). Clinical Course and Outcome of Human Monkeypox in Nigeria. Clin. Infect. Dis..

[36] Oakley L.P., Hufstetler K., O’Shea J., Sharpe J.D., McArdle C., Neelam V., Roth N.M., Olsen E.O., Wolf M., Pao L.Z. (2023). Mpox Cases Among Cisgender Women and Pregnant Persons—United States, May 11–November 7, 2022. MMWR Morb. Mortal. Wkly. Rep..

[37] García-Hernández L., Hernández-Aceituno A., Moreno Saavedra R.J., Larumbe-Zabala E. (2024). Case report: Clinical presentation of Monkeypox in pregnancy. Rev. Clin. Esp..

[38] Altindis M., Puca E., Shapo L. (2022). Diagnosis of monkeypox virus—An overview. Travel Med. Infect. Dis..

[39] Khattak S., Rauf M.A., Ali Y., Yousaf M.T., Liu Z., Wu D.D., Ji X.Y. (2023). The monkeypox diagnosis, treatments and prevention: A review. Front. Cell Infect. Microbiol..

[40] Brasil Ministério da Saúde (2022). Nota Técnica Nº, 44/2022-CGPAM/DSMI/SAPS/MS Recomendações Sobre Monkeypox no Ciclo Gravídico-Puerperal.

[41] Ministério da Saúde do Brasil (2024). Manual Monkeypox 2024: Guia Para Vigilância, Prevenção e Controle da Doença.

[42] World Health Organization (2022). Clinical Management and Infection Prevention and Control for Monkeypox: Interim Rapid Response Guidance.

[43] Health Protection Surveillance Centre (HPSC) (2022). Guidance for Tecovirimat: Monkeypox (TPOXX) Guidance Document v1.0.

[44] Yadav R., Chaudhary A.A., Srivastava U., Gupta S., Rustagi S., Rudayni H.A., Kashyap V.K., Kumar S. (2025). Mpox 2022 to 2025 update: A comprehensive review on its complications, transmission, diagnosis, and treatment. Viruses.

[45] Prévost J., Sloan A., Deschambault Y., Tailor N., Tierney K., Azaransky K., Kammanadiminti S., Barker D., Kodihalli S., Safronetz D. (2024). Treatment efficacy of cidofovir and brincidofovir against clade II monkeypox virus isolates. Antivir Res..

[46] Centers for Disease Control and Prevention (2025). Cidofovir Monograph. https://www.drugs.com/monograph/cidofovir.html.

[47] Schoenbaum S.C., Smith J., Brown R. (2024). Management of monkeypox in pregnancy. Ultrasound Obstet. Gynecol..

[48] (2024). Panel on guidelines for the prevention treatment of opportunistic infections in adults adolescents with HIV. Guidelines for the Prevention and Treatment of Opportunistic Infections in Adults and Adolescents with HIV.

[49] Andrieu J., Mège J.L., Mezouar S. (2025). Monkeypox virus and pregnancy. J. Med. Virol..

[50] Centers for Disease Control and Prevention (CDC) (2025). Monkeypox Vaccination.

[51] Fine P.E., Jezek Z., Grab B., Dixon H. (1988). The transmission potential of monkeypox virus in human populations. Int. J. Epidemiol..

[52] Centers for Disease Control and Prevention (CDC) (2024). Mpox Vaccine Effectiveness Data.

[53] Reynolds M.G., McCollum A.M., Nguete B., Shongo Lushima R., Petersen B.W. (2017). Improving the care and treatment of monkeypox patients in low-resource settings: Lessons learned from the Democratic Republic of the Congo. PLoS Negl. Trop. Dis..

[54] World Health Organization (WHO) (2022). Vaccines and Immunization for Monkeypox: Interim Guidance.

[55] Silva R.M.A., Kobayashi C.D., Martins A.F., Araújo A.C.M., Andrade P.H.S., Nóbrega M.E.B.D., Cabral C.M., Moraes M.B., Cardoso F.D., Victer T.N.D.F. (2024). Descriptive study of events allegedly attributable to mpox vaccination in Brazil in 2023. Cad. Saude Publica.

[56] Sanchez Clemente N., Coles C., Paixao E.S., Brickley E.B., Whittaker E., Alfven T., Rulisa S., Agudelo Higuita N., Torpiano P., Agravat P. (2024). Paediatric, maternal, and congenital mpox: A systematic review and meta-analysis. Lancet Glob. Health.

[57] Food and Drug Administration (FDA) (2023). JYNNEOS (Smallpox and Monkeypox Vaccine): Package Insert.

[58] Halsell J.S., Riddle J.R., Atwood J.E., Gardner P., Shope R., Poland G.A., Gray G.C., Ostroff S., Eckart R.E., Hospenthal D.R. (2003). Department of Defense Smallpox Vaccination Clinical Evaluation Team Myopericarditis following smallpox vaccination among vaccinia-naive USmilitary personnel. JAMA.

[59] Imran M., Sohail M., Kamran J., Abbas S.Q., Azeem K., Korir E. (2025). Vaccines and antiviral therapies for mpox virus in pregnant and breastfeeding women: Efficacy and maternal-child outcomes. Viruses.

[60] Cohn H., Bloom N., Cai G.Y., Clark J.J., Tarke A., Bermúdez-González M.C., Altman D.R., Lugo L.A., Lobo F.P., Marquez S. (2023). Mpox vaccine and infection-driven human immune signatures: An immunological analysis of an observational study. Lancet Infect. Dis..

[61] Centers for Disease Control and Prevention (2025). Mpox Monitoring and Risk Assessment for People Exposed.

[62] Centers for Disease Control Prevention (CDC) (2024). Isolation Infection Control for Home: Mpox.

[63] Poland G.A., Kennedy R.B., Tosh P.K. (2022). Prevention of monkeypox with vaccines: A rapid review. Lancet Infect. Dis..

[64] Pan American Health Organization (PAHO) (2023). Guidance on the Use of Mpox Vaccines.

[65] Dubey T., Chakole S., Agrawal S., Gupta A., Munjewar P.K., Sharma R., Yelne S. (2023). Enhancing Nursing Care in Monkeypox (Mpox) Patients: Differential Diagnoses, Prevention Measures, and Therapeutic Interventions. Cureus.

[66] Philpott D., Hughes C.M., Alroy K.A., Kerins J.L., Pavlick J., Asbel L., Crawley A., Newman A.P., Spencer H., Feldpausch A. (2022). Epidemiologic and Clinical Characteristics of Monkeypox Cases—United States, May 17–July 22, 2022. MMWR Morb. Mortal. Wkly. Rep..

[67] Kava C.M., Rohraff D.M., Wallace B., Mendoza-Alonzo J.L., Currie D.W., Munsey A.E., Roth N.M., Bryant-Genevier J., Kennedy J.L., Weller D.L. (2022). Epidemiologic Features of the Monkeypox Outbreak and the Public Health Response—United States, May 17–October 6, 2022. MMWR Morb. Mortal. Wkly. Rep..

[68] Angelo K.M., Smith T., Camprubí-Ferrer D., Balerdi-Sarasola L., Díaz Menéndez M., Servera-Negre G., Barkati S., Duvignaud A., Huber K.L.B., Chakravarti A. (2023). Epidemiological and clinical characteristics of patients with monkeypox in the GeoSentinel Network: A cross-sectional study. Lancet Infect. Dis..

[69] Miller M.J., Cash-Goldwasser S., Marx G.E., Schrodt C.A., Kimball A., Padgett K., Noe R.S., McCormick D.W., Wong J.M., Labuda S.M. (2022). Severe Monkeypox in Hospitalized Patients—United States, August 10–October 10, 2022. MMWR Morb. Mortal. Wkly. Rep..

